# Genetic and biological characteristics of avian influenza virus subtype H1N8 in environments related to live poultry markets in China

**DOI:** 10.1186/s12879-019-4079-z

**Published:** 2019-05-22

**Authors:** Ye Zhang, Jie Dong, Hong Bo, Libo Dong, Shumei Zou, Xiyan Li, Yuelong Shu, Dayan Wang

**Affiliations:** 1 Chinese National Influenza Centre, National Institute for Viral Disease Control and Prevention, Chinese Center for Disease Control and Prevention; WHO Collaborating Center for Reference and Research on Influenza; Key Laboratory for Medical Virology, National Health and Family Planning Commission, Beijing, China; 2 Present Address: Public Health School (Shenzhen), Sun Yat-sen University, Guangzhou, China

**Keywords:** Avian influenza virus, H1N8 subtype, Live poultry market

## Abstract

**Background:**

Since 2008, avian influenza surveillance in poultry-related environments has been conducted annually in China. Samples have been collected from environments including live poultry markets, wild bird habitats, slaughterhouses, and poultry farms. Multiple subtypes of avian influenza virus have been identified based on environmental surveillance, and an H1N8 virus was isolated from the drinking water of a live poultry market.

**Methods:**

Virus isolation was performed by inoculating influenza A-positive specimens into embryonated chicken eggs. Next-generation sequencing was used for whole-genome sequencing. A solid-phase binding assay was performed to test the virus receptor binding specificity. Trypsin dependence plaque formation assays and intravenous pathogenicity index tests were used to evaluate virus pathogenicity in vitro and in vivo, respectively. Different cell lines were chosen for comparison of virus replication capacity.

**Results:**

According to the phylogenetic trees, the whole gene segments of the virus named A/Environment/Fujian/85144/2014(H1N8) were of Eurasian lineage. The HA, NA, PB1, and M genes showed the highest homology with those of H1N8 or H1N2 subtype viruses isolated from local domestic ducks, while the PB2, PA, NP and NS genes showed high similarity with the genes of H7N9 viruses detected in 2017 and 2018 in the same province. This virus presented an avian receptor binding preference. The plaque formation assay showed that it was a trypsin-dependent virus. The intravenous pathogenicity index (IVPI) in chickens was 0.02. The growth kinetics of the A/Environment/Fujian/85144/2014(H1N8) virus in different cell lines were similar to those of a human-origin virus, A/Brisbane/59/2007(H1N1), but lower than those of the control avian-origin and swine-origin viruses.

**Conclusions:**

The H1N8 virus was identified in avian influenza-related environments in China for the first time and may have served as a gene carrier involved in the evolution of the H7N9 virus in poultry. This work further emphasizes the importance of avian influenza virus surveillance, especially in live poultry markets (LPMs). Active surveillance of avian influenza in LPMs is a major pillar supporting avian influenza control and response.

**Electronic supplementary material:**

The online version of this article (10.1186/s12879-019-4079-z) contains supplementary material, which is available to authorized users.

## Background

Both animal and human infections with influenza A virus have been reported. Sometimes mammals, such as pigs, horses, and seals, and poultry can be infected under natural conditions. Wild aquatic birds are the major reservoir of avian influenza, harbouring 16 haemagglutinin (HA) and 9 neuraminidase (NA) subtypes of viruses [1]. Although avian influenza viruses present limited replication ability in humans, direct human infection with avian influenza and pandemics caused by reassortment of human influenza have both occurred. To date, human infections have been reported with the H5, H6, H7, H9, and H10 influenza virus subtypes [2, 3].

Exposure to live poultry markets (LPMs) is an important risk factor for highly pathogenic avian influenza infection. Closure of LPMs has been reported to be efficient in blocking avian influenza transmission [4, 5]. In China, LPMs are major places enabling influenza dissemination and potential reassortment because of the high densities and the mixture of poultry, pet birds, and wild birds that are often present in LPMs. LPMs are also considered valuable places for influenza ecology research and research on the emergence and re-emergence of influenza type A virus [6]. Therefore, avian influenza surveillance in LPMs provides not only clues for tracing infection sources but also evidence for risk assessment and decision making [7–9].

Along with avian influenza control and prevention, regular LPM surveillance has been conducted in China annually since 2008. Specimens are sampled from relevant environments, and viruses are isolated and identified. The distribution and prevalence of influenza A virus subtypes in LPMs are then analysed. Multiple subtypes of avian influenza virus have been identified in the more than 10 years of surveillance. One avian influenza virus subtype, H1N8, which is uncommon among animals and animal-associated environments, was identified from poultry drinking water in a LPM in Fujian Province in 2014. To better understand its potential risk to human health, studies were conducted on the genetic and biological characteristics of the virus.

## Methods

### RNA extraction and real-time RT-PCR

A QIAamp Viral RNA Mini Kit (Qiagen, Hilden, Germany) was used to carry out RNA extraction. Real-time reverse transcription polymerase chain reaction (RT-PCR) assays for the influenza A were performed on each of the samples. The reactions were carried out using an AgPath-ID™ One-Step RT-PCR Kit (ThermoFisher, Waltham, USA) under the following conditions: 10 min at 45 °C; 10 min at 95 °C; and 40 cycles of 15 s at 95 °C and 45 s at 60 °C. The sequences of the primers and probe are as follows: forward primer, 5’GACCRATCCTGTCACCTCTGAC3’; reverse primer, 5’AGGGCATTYTGGACAAAKCGTCTA3’; and probe, 5’FAM-TGCAGTCCTCGCTCACTGGGCACG-BHQ1–3′.

### Virus amplification

Viruses or influenza A-positive specimens were inoculated into the allantoic cavity of 9-day-old embryonated chicken eggs for 48 h at 37 °C for virus propagation. The allantoic fluid was harvested, and haemagglutination assays were performed using 1% turkey red blood cells to detect the titres of the influenza viruses.

### Full-genome sequencing

PathAmp FluA Reagents (ThermoFisher, Waltham, USA) were used to amplify the full genome. The primer sequences were as follows: 5′-CTGGATACGCCAGCRAAAGCAGG-3′ (sense) and 5′-GACCTGATGCGGAGTAGAAACAAGG-3′ (antisense). Whole-genome sequencing was then performed on an Ion Torrent™ Personal Genome Machine™ platform (Thermo Fisher Scientific) with a read length of 200 base pairs following the instructions, and the data were analysed using CLC Genomics Workbench 7.5.1 software.

### Phylogenetic analysis

Phylogenetic analysis was performed using MEGA7.0, and a phylogenetic tree was constructed using the maximum likelihood method. The bootstrap value was tested with 1000 replications for each gene segment. Homology analysis of nucleic acids was performed on the NCBI website with the BLAST platform.

### Receptor binding analysis

A solid-phase binding assay was used as described previously [10, 11]. Synthetic sialylglycopolymers, including 3′-SLN, 6′-SLN, 3′-SL, and 6′-SL (referring to Neu5Acα2, 3Galβ1-4GlcNAc, Neu5Acα2,6Galβ1-4GlcNAc, Neu5Acα2,3Galβ1-4Glc, and Neu5Acα2,6Galβ1-4Glc, respectively), were coated on the plates. For testing, 32 haemagglutination units (HAUs) of each virus were added to each well. ELISA was then performed. The primary antibody was a universal monoclonal antibody against group I HAs, and a goat anti-human IgG-HRP antibody was chosen as the secondary antibody. A tetramethylbenzidine (TMB) substrate solution (BD Biosciences) was used to develop the results. The optical density was read at 450 nm.

### Trypsin dependence plaque formation assay

Virus pathogenicity in vitro can be reflected by plaque formation with or without trypsin. The plaque formation protocol was performed as follows: 96-well MDCK plates with 3 × 10^4^ cells/well were cultured at 37 °C overnight. Serial dilutions of virus were inoculated into MDCK cells. Two hours after virus absorption, overlay medium (2× DMEM and Avicel) with or without trypsin (final concentration of 2 μg/ml) was prepared and added. The cell plates were fixed with 4% paraformaldehyde 1 day later. ELISA was performed with a mouse monoclonal antibody against influenza type A (CDC-WHO kit used at 1:2000 in ELISA buffer) as the primary antibody and a goat anti-mouse IgG (H + L) HRP conjugate (Bio-Rad 172–1011, used at 1:1000 in ELISA Buffer) as the secondary antibody. Finally, plaque formation was visualized by adding True Blue™ peroxidase substrate (KPL 50–78-02) and 0.03% H_2_O_2_ (1:1000 dilution of a 30% solution) [12].

### Intravenous pathogenicity index test in chicken

Five-week-old specific pathogen-free (SPF) leghorn chickens were purchased from the company Beijing Vital River Laboratory Animal Technology. According to the OIE/WHO guidelines [13], the chickens were divided into two groups. One group with 10 chickens was infected intravenously with 100 μl of diluted test virus (fresh allantoic fluid diluted 1:10 with sterile isotonic saline). The other group, with 2 chickens, was infected intravenously with 100 μl of isotonic saline. The chickens were observed for 10 days.

### Virus replication kinetics in DF1, A549 and PK15 cells

The virus replication capacity in mammalian cells was evaluated. Human type II alveolar epithelial cell line (A549), chicken embryo fibroblasts cell line DF1 (DF1), and porcine kidney cell line (PK15) were inoculated into 96-well plates at a density of 3 × 10^4^ cells/well at 37 °C overnight, and the test viruses were added with a multiplicity of infection of 0.01. The plates were then incubated with DMEM containing 0.5% BSA and 2 μg/ml TPCK-treated trypsin at 37 °C. The supernatants were collected at 12, 24, 36, 48, 60 and 72 h post infection. End-point titration of the supernatants was performed with MDCK cells.

### Statistical analysis

The statistical significance of the differences was determined using T tests, and *P* < 0.05 was considered to indicate a significant difference.

## Results

### Homology analysis

The avian influenza virus subtype H1N8 identified in this study was named A/Environment/Fujian/85144/2014(H1N8); the full genome sequences of the virus have been uploaded to the Global Initiative on Sharing All Influenza Database (GISAID) with accession numbers EPI11315724-EPI11315731. The nucleotide homology analysis of A/Environment/Fujian/85144/2014 showed that it might be a reassortant virus. Three of the 8 segments, HA, NA and PB2, were derived from an A/Duck/Fujian/13/2013 (H1N8)-like virus; the PB2, PA, and NS genes showed the highest similarity with the H7N9 virus A/Environment/Fujian/S10058/2017(H7N9). NP and M were most closely related to the H1N2 viruses A/Environment/Fujian/FJ1273/2014 and A/duck/Guangxi/GXd-2/2012, respectively (Table 1).

**Table 1:** Nucleotide homology of the A/Environment/Fujian/85144/2014 (H1N8) virus with the most closely related strains in GenBank Database

Gene segment	Virus with the highest nucleic acid homology	Percent Homology (%)	GenBank ID
PB2	A/environment/Fujian/S10058/2017(H7N9)	99	MH209528.1
PB1	A/duck/Fujian/13/2013(H1N8)	99	KP658098.1
PA	A/environment/Fujian/S10058/2017(H7N9)	99	MH209530.1
HA	A/duck/Fujian/13/2013(H1N8)	99	KP658038.1
NP	A/environment/Fujian/FJ1273/2014(H1N2)	99	KP658061.1
NA	A/duck/Fujian/13/2013(H1N8)	99	KP658023.1
M	A/duck/Guangxi/GXd-2/2012(H1N2)	99	KF013935.1
NS	A/environment/Fujian/S10058/2017(H7N9)	99	MH209535.1

### Molecular characteristics

The HA cleavage site of the H1N8 subtype virus had a PSVQSR/GLF motif, which presented low pathogenic avian influenza properties. The Q226L or G228S mutation in HA, which is related to human-type receptor binding preference, was not detected in the H1N8 virus in this study. The E627K and D701N substitutions (molecular markers related to increased virulence in mammals [14]) of the PB2 protein were not detected. The N30D and T215A substitutions of the M1 protein were found in the H1N8 virus, and this mutation has been reported to be associated with increased pathogenicity of the H5N1 virus in mice [15]. No neuraminidase inhibitor or amantadine resistance mutations [16] were found in the NA protein of the H1N8 virus. The NS1 amino acid at position 42 was A instead of S, which indicated that the P42S substitution related to increased virulence in mice [17] was not present in the H1N8 virus (Table 2).

**Table 2:** Key molecular markers of the A/Environment/Fujian/85144/2014 (H1N8) virus

Function	Amino- Acid Mutation	Molecular characteristics of A/Environment/Fujian/85144/2014(H1N8)
HA		
HA cleavage		PSVQSR/GLF
Human receptor binding(H3 numbering) [14]	Q226L	Q
	G228S	G
NA		
Resistant to oseltamivir or zanamivir (N2 numbering) [15]	R292K	R
	H274Y	H
	E119V	E
	R152K	R
PB2		
Increased virulence in mice [16]	E627K	E
Enhances pathogenicity in mice [16]	D701N	D
M1		
Increased virulence in mice [17]	N30D	D
	T215A	A
M2		
Resist to amantadine	S31 N	S
NS1		
Increased virulence in mice [18]	P42S	A

### Phylogenetic relationship

**Fig. 1:**
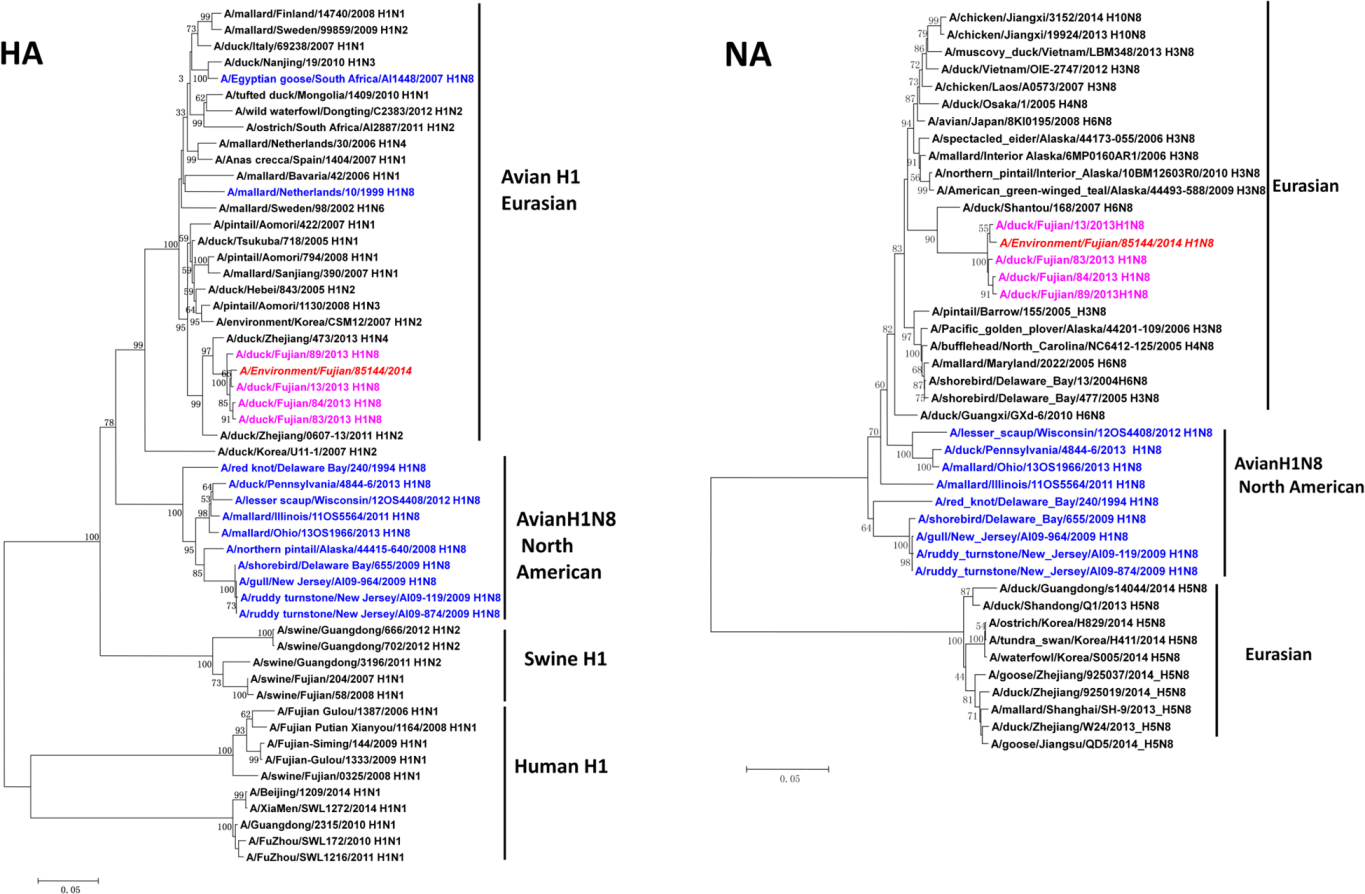
Phylogenetic trees of the HA and NA genes. The tree was created by the maximum likelihood method and bootstrapped with 1000 replicates using MEGA7 software. The scale bar represents the distance unit between sequence pairs. Blue represents avian influenza H1N8, pink represents avian influenza isolates from China, and red represents the A/Environment/Fujian/85144/2014 (H1N8) virus in this study

Phylogenetic analysis indicated that the HA gene of A/Environment/Fujian/85144/2014(H1N8) was clustered with those of H1N8 viruses isolated from local ducks in Fujian Province named A/duck/Fujian/13/2013(H1N8), A/duck/Fujian/83/2013(H1N8), A/duck/Fujian/89/2013(H1N8) and A/duck/Fujian/84/2013(H1N8). These viruses belong to the avian H1 group of the Eurasian lineage; they are different from the avian H1N8 North American–lineage viruses and phylogenetically far from swine–origin H1 and seasonal human H1 subtype viruses. The N8 gene evolutionary tree indicated that the studied H1N8 virus was most likely derived from the local duck H1N8 viruses and clustered with the duck H6N8 virus isolated from Shantou, Guangdong Province. The N8 gene in the studied virus was clearly different from the N8 genes of avian influenza H1N8 North American–lineage viruses (Fig. 1). According to the phylogenetic analyses of six internal genes, the studied A/Environment/Fujian/85144/2014(H1N8) virus belongs to the Eurasian lineage and shows phylogenetic diversity. The PB2, PA, NP and NS genes were clustered with those of H7N9 viruses detected in the environment or isolated from ducks in 2017 and 2018 (Additional file 1: Figure S1).

### Growth kinetics of the H1N8 virus in DF1, A549 and PK15 cells

**Fig. 2:**
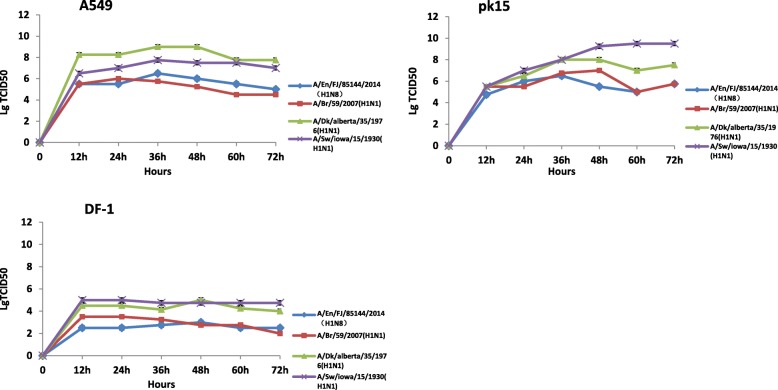
Growth kinetics of different viruses in the A549, PK15 and DF1 cell lines A/Environment/Fujian/85144/2014, A/Brisbane/59/2007, A/duck/Alberta/1976, and A/swine/15/1930 were inoculated into the A549, DF1, and PK15 cell lines, respectively. The multiplicity of infection was 0.01. The supernatants were collected at 0, 12, 24, 36, 48, 60 and 72 h. The log_10_TCID_50_ was used to evaluate the viral load

The growth kinetics of A/Environment/Fujian/85144/2014 were compared with those of A/Brisbane/59/2007(H1N1), A/duck/Alberta/35/1976(H1N1), and A/swine/Iowa/15/1930(H1N1), which are of human origin, avian origin, and swine origin, respectively. The mean peak titres of A/Environment/Fujian/85144/2014, A/Brisbane/59/2007 (H1N1), A/duck/Alberta/35/1976(H1N1), and A/swine/Iowa/15/1930(H1N1) in A549 cells were 6, 5.25, 9 and 7.5 log_10_TCID_50_/100 μl, respectively, while those in the PK15 cell line were 5.5, 7, 8 and 9.25 log_10_TCID_50_/100 μl, respectively; however, in the DF1 cell line, the values were 3, 2.75, 4.15 and 4.75 log_10_TCID_50_/100 μl, respectively. All 4 selected viruses with different origins presented higher titres in A549 and PK15 cells than in DF1 cells (*P* < 0.05). The growth of the A/Environment/Fujian/85144/2014(H1N8) virus in the different cell lines was similar to that of the human-origin virus A/Brisbane/59/2007(H1N1) and lower than that of the control avian-origin and swine-origin viruses, A/duck/Alberta/35/1976(H1N1) and A/swine/Iowa/15/1930(H1N1), respectively (Fig. 2).

### Plaque formation assay

**Fig. 3:**
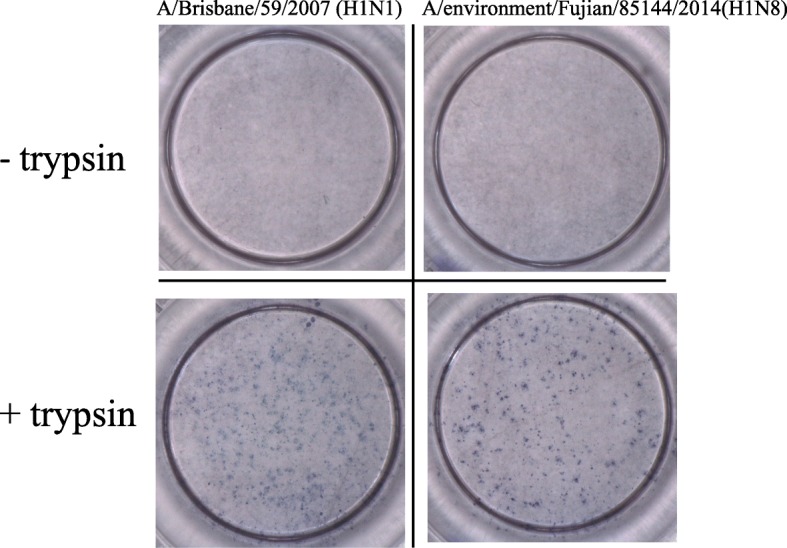
Plaque formation in MDCK cells with or without TPCK-treated trypsin. A/Environment/Fujian/85144/2014(H1N8) and A/Brisbane/59/2007(H1N1) were inoculated into MDCK cells with or without TPCK-treated trypsin (2 μg/ml) at 37 °C overnight. Both viruses produced clear plaques with trypsin present, while no plaques were produced without trypsin

No plaques formed when A/Environment/Fujian/85144/2014(H1N8) or the reference seasonal influenza virus A/Brisbane/59/2007(H1N1) was inoculated into MDCK cells without addition of TPCK-treated trypsin. However, clear plaques of both viruses were present when trypsin was added, and the sizes and shapes of the plaques were similar. This result showed that the studied H1N8 virus is a low-pathogenic avian influenza virus in vitro (Fig. 3).

### Receptor binding specificity

**Fig. 4:**
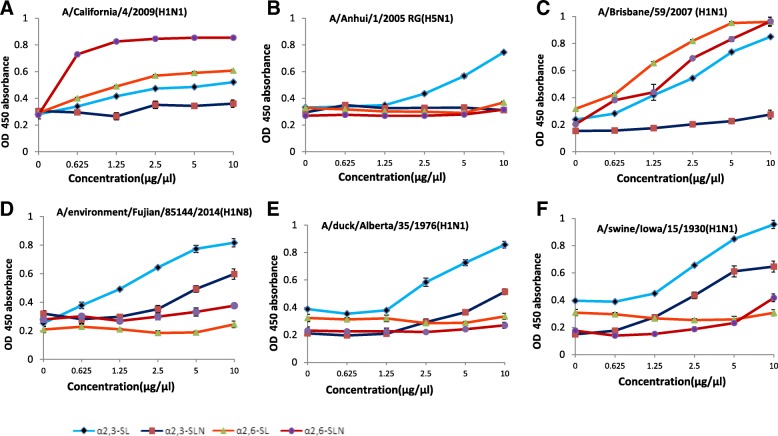
Characterization of receptor binding properties. Four different sialylglycopolymers (light blue line with diamonds, 2,3-SL; dark blue line with squares, α2,3-SLN; orange line with triangles, α2,6-SL; red line with circles, α2,6-SLN) were coated onto plates and bound with different viruses. **a**, **b**, **c**, **d**, **e**, and **f** Represent the binding properties of A/California/4/2009(H1N1), A/Anhui/1/2005RG(H5N1), A/Brisbane/59/2007(H1N1), A/Environment/Fujian/85144/2014(H1N8), A/duck/Alberta/35/1976(H1N1) and A/swine/Iowa/15/1930(H1N1), respectively. The data shown are the mean ± SD

A solid-phase binding assay was used to test six viruses, including 5 reference viruses with different origins. Four different types of sialylglycopolymers (α2,3-SL, α2,6-SL, α2,3-SLN and α2,6-SLN) were coated on the plates at concentrations of 0, 0.625, 1.25, 2.5, 5 and 10 μg/ml [18]. As shown in Fig. 4, A/California/4/2009(H1N1) bound α2,6-SLN, A/Anhui/1/2005RG(H5N1) bound α2,3-SL, and A/Brisbane/59/2007(H1N1) bound α2,3-SL, α2,6-SL and α2,6-SLN. All three H1 subtype viruses, A/Environment/Fujian/85144/2014(H1N8), A/duck/Alberta/35/1976(H1N1) and A/swine/Iowa/15/1930(H1N1), bound α2,3-SL (Fig. 4). The results showed that the reference human H1N1 virus A/California/4/2009(H1N1) had a human receptor binding preference, A/Brisbane/59/2007(H1N1) presented dual receptor binding properties, and H5N1 had avian receptor binding characteristics, consistent with previous reports. Our study indicated that all three tested H1 subtype viruses, including A/Environment/Fujian/85144/2014(H1N8), exhibited avian receptor binding preferences.

### Intravenous pathogenicity index test in chickens

During the 10-day observation period, only 2 chickens presented mild signs and cough as early as 2 days after infection. The other chickens, including those in the test and control groups, appeared normal until the end of observation. The IVPI in the chickens was 0.02, which indicated that A/Environment/Fujian/85144/2014(H1N8) is a low-pathogenic avian influenza virus.

## Discussion

H1 subtype influenza viruses are known for their wide range of hosts (from birds to mammalian species such as pigs and humans) and rapid evolution as a result of antigenic drift and antigenic shift, which can cause seasonal influenza epidemics and even influenza pandemics [1, 19, 20]. The phylogenetic analysis showed that H1 subtype influenza viruses have distinct lineages that can be separated based on avian, human and swine origins. Cross-species transmission events of H1 subtype influenza virus have happened occasionally [21–23]. Our study showed that the H1N8 avian influenza virus A/Environment/Fujian/85144/2014(H1N8) is derived from avian-origin viruses and is evolutionarily distant from mammalian viruses. Although avian-origin influenza viruses are distant from mammalian ones, they still make genomic contributions to the evolution of mammalian viruses [24]. We should be vigilant about the potential emergence of pandemics due to avian influenza H1 subtype viruses.

Research on avian influenza virus ecology in LPMs in China has demonstrated that H5, H9, and H7 are not the only subtypes of avian influenza viruses; subtypes such as H3, H4, H6, H1, and H2 are also present [6, 25]. A few studies have indicated that H1N2, H1N1, and H1N3 are relatively easy to detect, although both H1N4 and H1N9 had been isolated as well [26, 27]. To our knowledge, the H1N8 subtype has rarely been detected [28, 29]. Our study may be the first systematic study of the biological and genetic characteristics of the H1N8 avian influenza virus, which was isolated from live poultry market-related environments in China.

Receptor binding preference plays an important role in influenza replication and transmission. The ability of avian influenza virus to bind to the human receptor is the basis for efficient human-to-human transmission. The relationship between human receptor binding specificity and HA gene molecular markers has been extensively revealed in H5, H7 and H9 subtype viruses [22, 30]. Based on the solid-phase binding assay results, the studied H1N8 virus still has an avian receptor binding preference.

Plaque formation assays are some of the most quantitative and useful biological methods for virus research and can be used for quantification of infectivity and identification of individual virus particles with specific biological features. We intended to determine the pathogenicity of the H1N8 avian influenza virus in vitro using this assay. If a virus is highly pathogenic, a plaque can be formed without trypsin present. This property is based on the presence of multiple basic amino acids at the cleavage site of the HA protein [31]. We found that the H1N8 virus needed trypsin to form plaques, which indicated its low-pathogenic avian influenza characteristics. This phenotype is supported by the sequence of the HA cleavage site, PSVQSR/GLF, which has only one basic amino acid.

Virus growth curves in different cell lines can be used to describe virus replication and proliferation ability. In our study, the H1N8 virus grew better in A549 and PK15 cells than in DF1 cells, which indicated the possibility of a growth preference for mammalian cells. A sharp increase in virus replication was detected 12 h post infection in the A549 and DF1 cells. However, the virus growth in PK15 cells increased to a peak at 36 h post infection. A/Environment/Fujian/85144/2014 displayed avian receptor binding characteristics, indicating that the virus retains avian influenza binding capacity. Despite its great similarity with other avian influenza viruses and its low pathogenicity to poultry, this H1N8 subtype avian influenza virus presented efficient replication in mammalian cells, indicating its potential risk for poultry and even mammals. Further exploration will be beneficial to enhance understanding of the mechanism of H1N8 infection in birds and mammals.

The PB2, PA, and NS genes of the H1N8 virus studied showed the highest similarity with a highly pathogenic avian influenza (HPAI) H7N9 virus, A/Environment/Fujian/S10058/2017(H7N9), which was reported in 2018 [32]. Although the H1N8 NP gene was most similar to that of the H1N2 virus, it shared high similarity and clustered with those of the H7N9 viruses detected in 2017 and 2018. These HPAI H7N9 viruses were detected in poultry in China [32] and did not present the same gene constellations as HPAI H7N9 viruses possessing internal genes from H9N2 viruses that crossed species barriers and caused sporadic human infections [11]. Our study showed that the same genetic pool (the PB2, PA, NP, and NS genes) that circulated in 2014 was still present in 2017, indicating that A/Environment/Fujian/85144/2014(H1N8) acted as a gene carrier and was involved in the evolution of H7N9 viruses in poultry. Active surveillance of avian influenza in LPMs is a major pillar supporting avian influenza control and response.

## Conclusions

The H1N8 influenza virus in this study contained a gene constellation of avian origin and might have been involved in the evolution of H7N9 viruses in poultry. In addition, an avian receptor binding preference was present, and low pathogenicity to poultry was confirmed both in vitro and in vivo. This H1N8 virus can also grow well in mammalian cells. Although this subtype of virus is not frequently detected, it still contributes to the diversity of avian influenza ecology and provides some insights for virus surveillance. The results of this study also emphasize the forecasting function of LPMs regarding the trends in avian influenza activity in China.

## Additional file

Additional file 1: **Figure S1.** Phylogenetic analysis of six internal genes of A/Environment/Fujian/85144/2014(H1N8). Phylogenetic analysis of six internal genes, including PB2, PB1, PA, NP, M, and NS. All sequences were downloaded from GenBank. The maximum likelihood method was used to construct the phylogenetic trees. The virus strains are marked with different colours. Red represents the A/Environment/Fujian/85144/2014(H1N8) strain in this study, blue represents other H1N8 subtype viruses, pink represents all available previous H1N8 subtype viruses sequence from China, black represents viruses of other subtypes, and the black star indicates the HPAI H7N9 viruses isolated in 2017 and 2018. (PDF 1538 kb)

## Abbreviations

A549: Human type II alveolar epithelial cell line
DF1: DF1 Chicken embryo fibroblasts cell line
HA: Haemagglutinin
IVPI: Intravenous pathogenicity index
LPM: Live poultry market
NA: Neuraminidase
PK15: Porcine Kidney cell line
SPF: Specific pathogen free
TCID_50_: 50% Tissue culture infective dose
TPCK: 6- (1-tosylamido-2-phenyl) ethyl chloromethyl ketone
TRBC: Turkey red blood cell
