# Supplementary figures and images for: Genetic and biological characteristics of avian influenza virus subtype H1N8 in environments related to live poultry markets in China
Source: BMC Infect Dis. 2019 May 22;19:458. doi: 10.1186/s12879-019-4079-z (PMC6532177; doi:10.1186/s12879-019-4079-z)

PB2

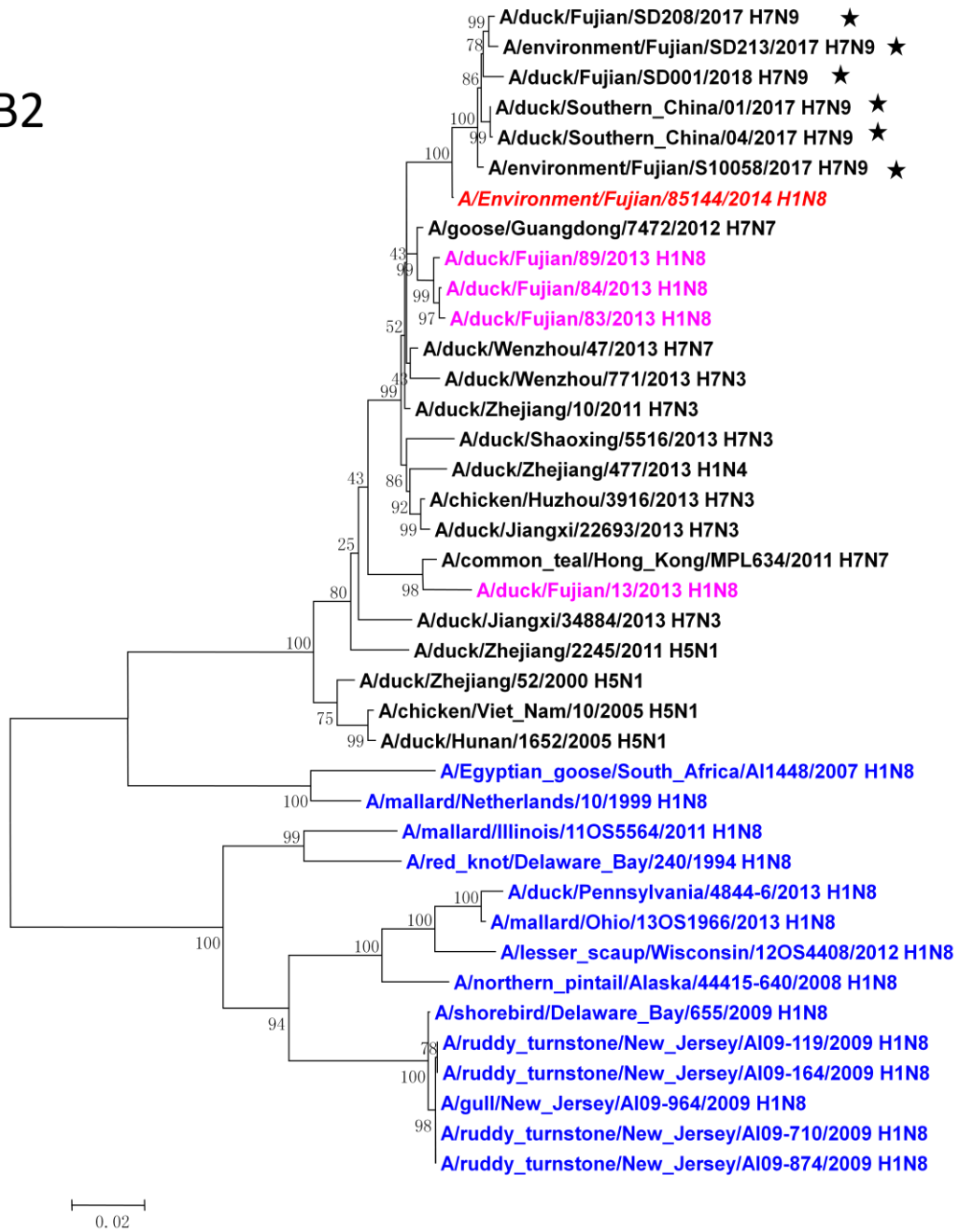

PB1

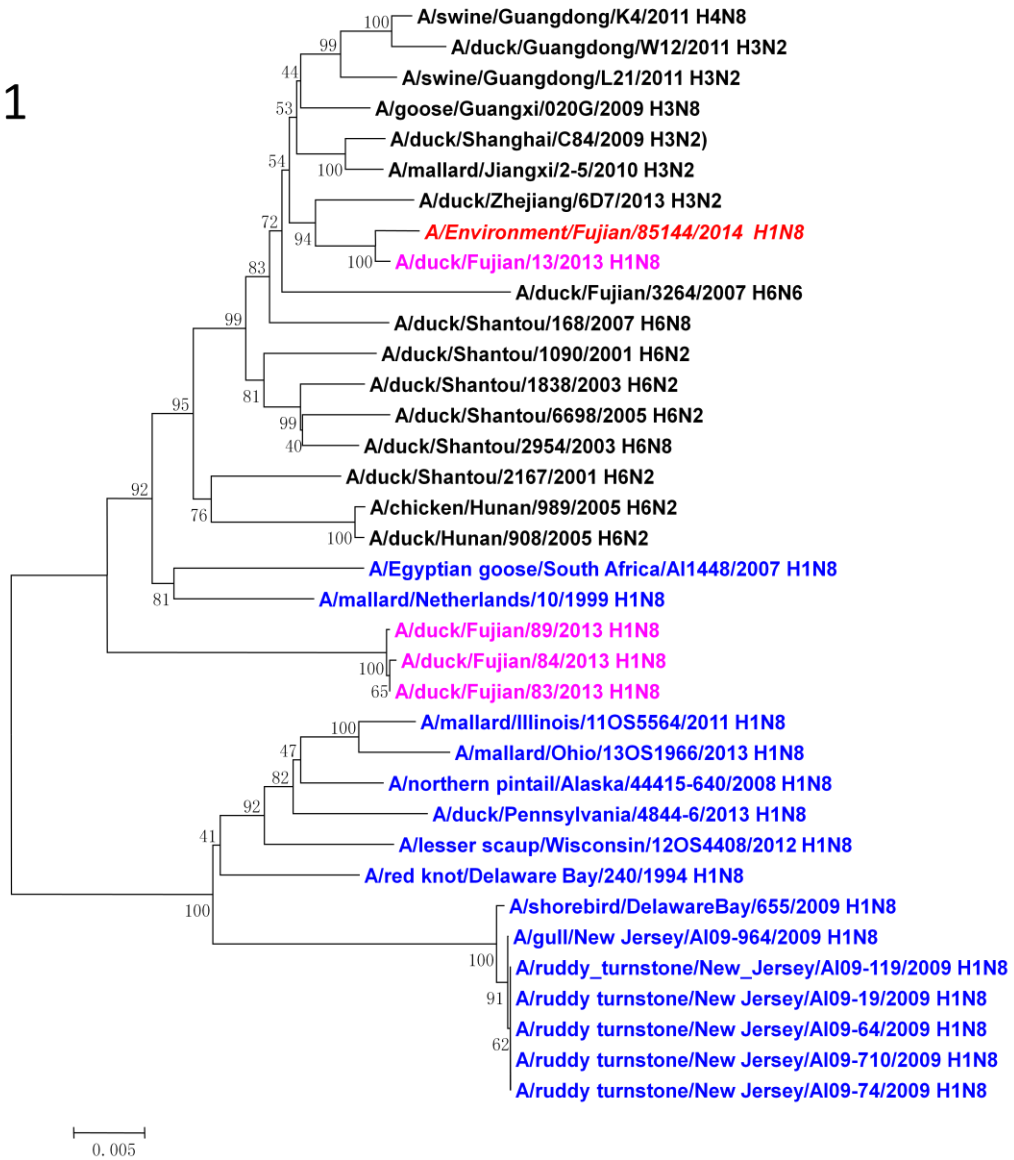

PA

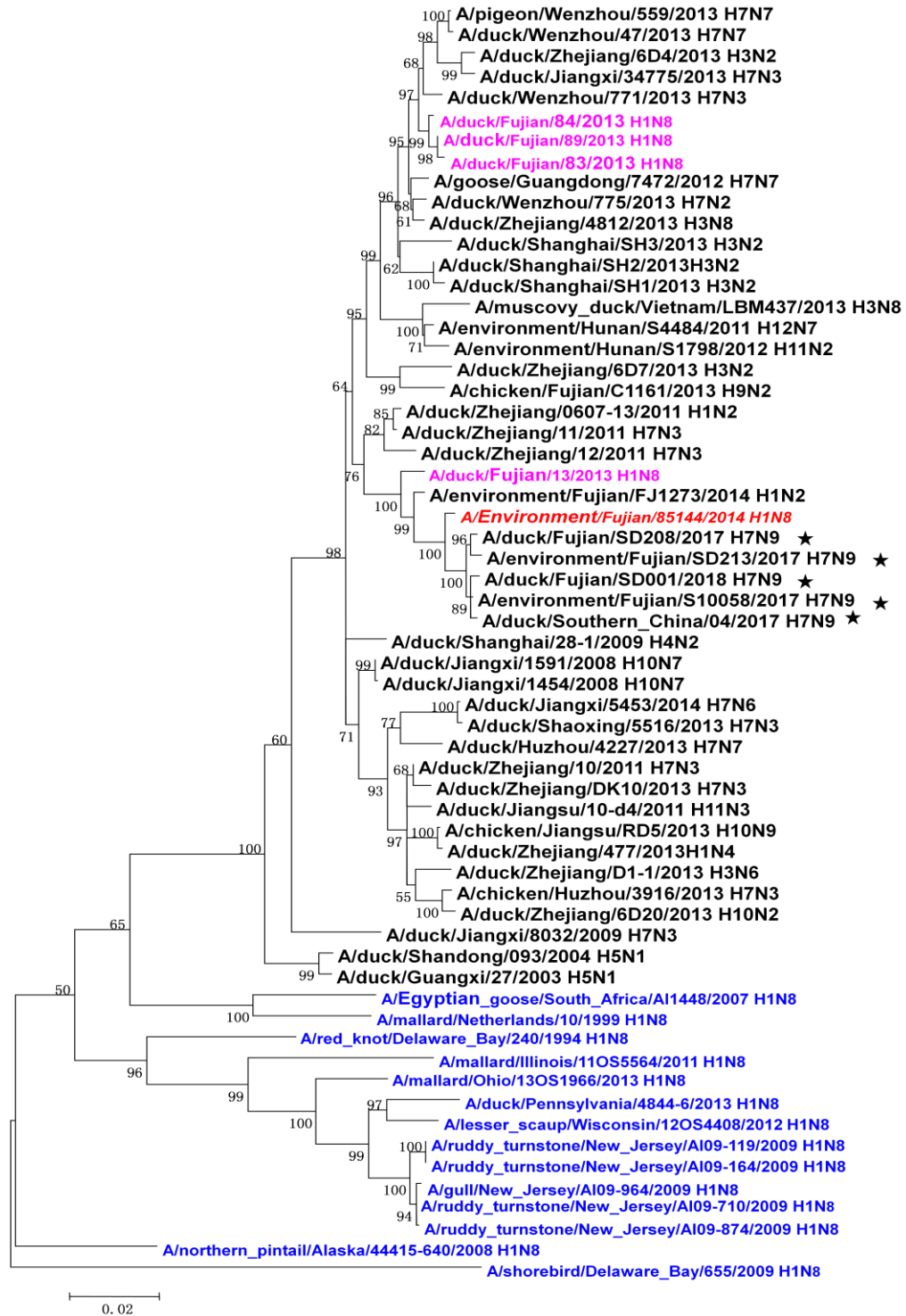

NP

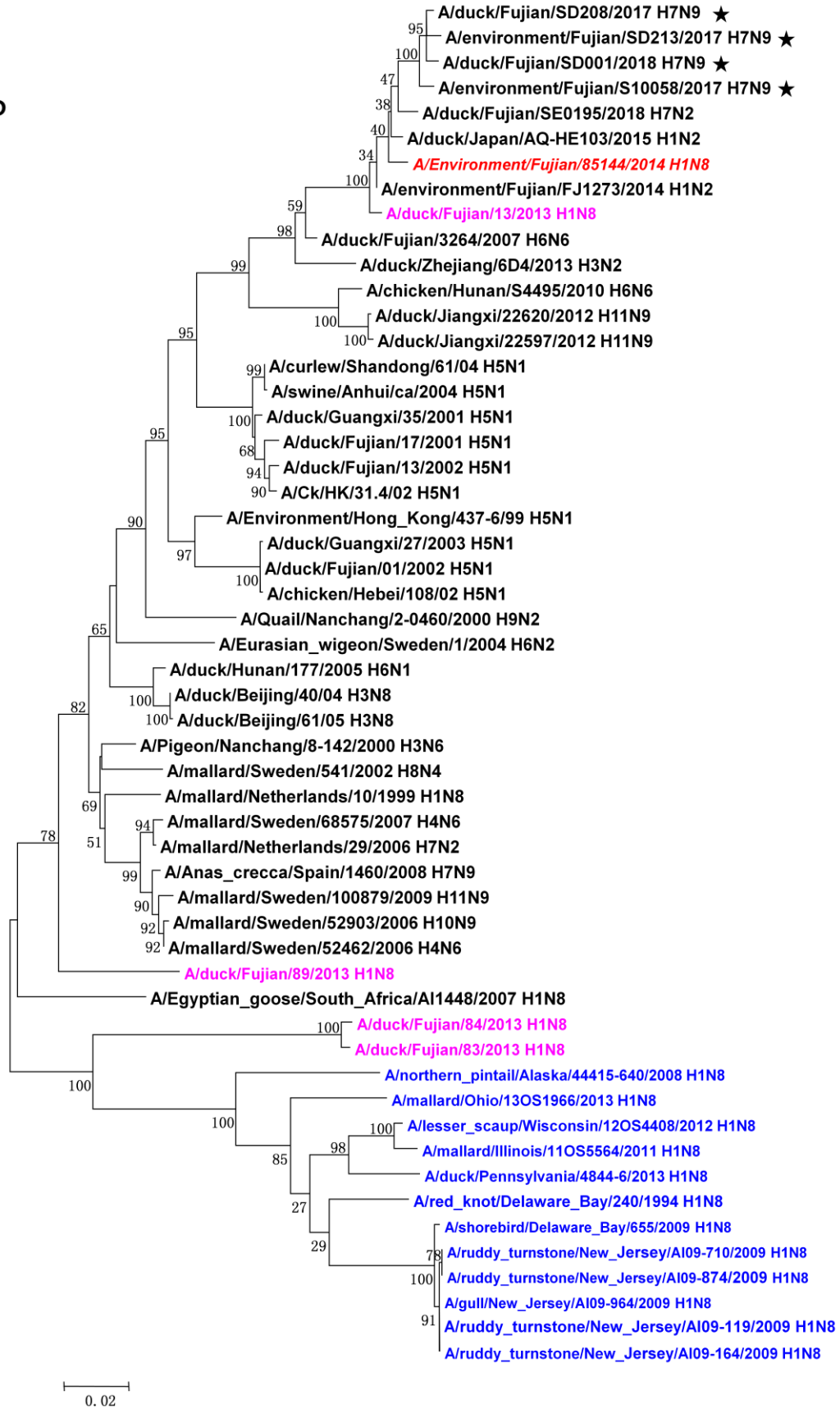

M

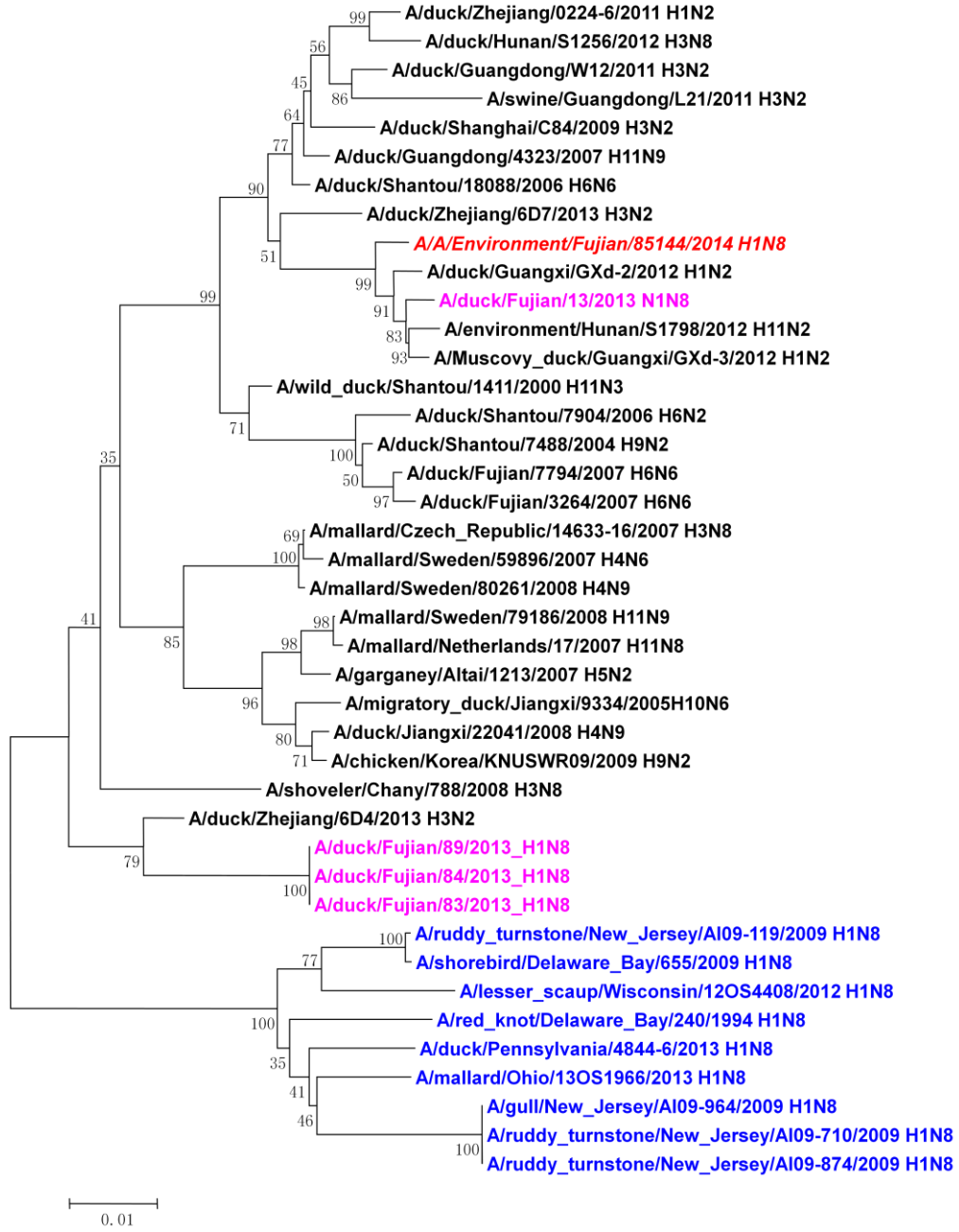

NS

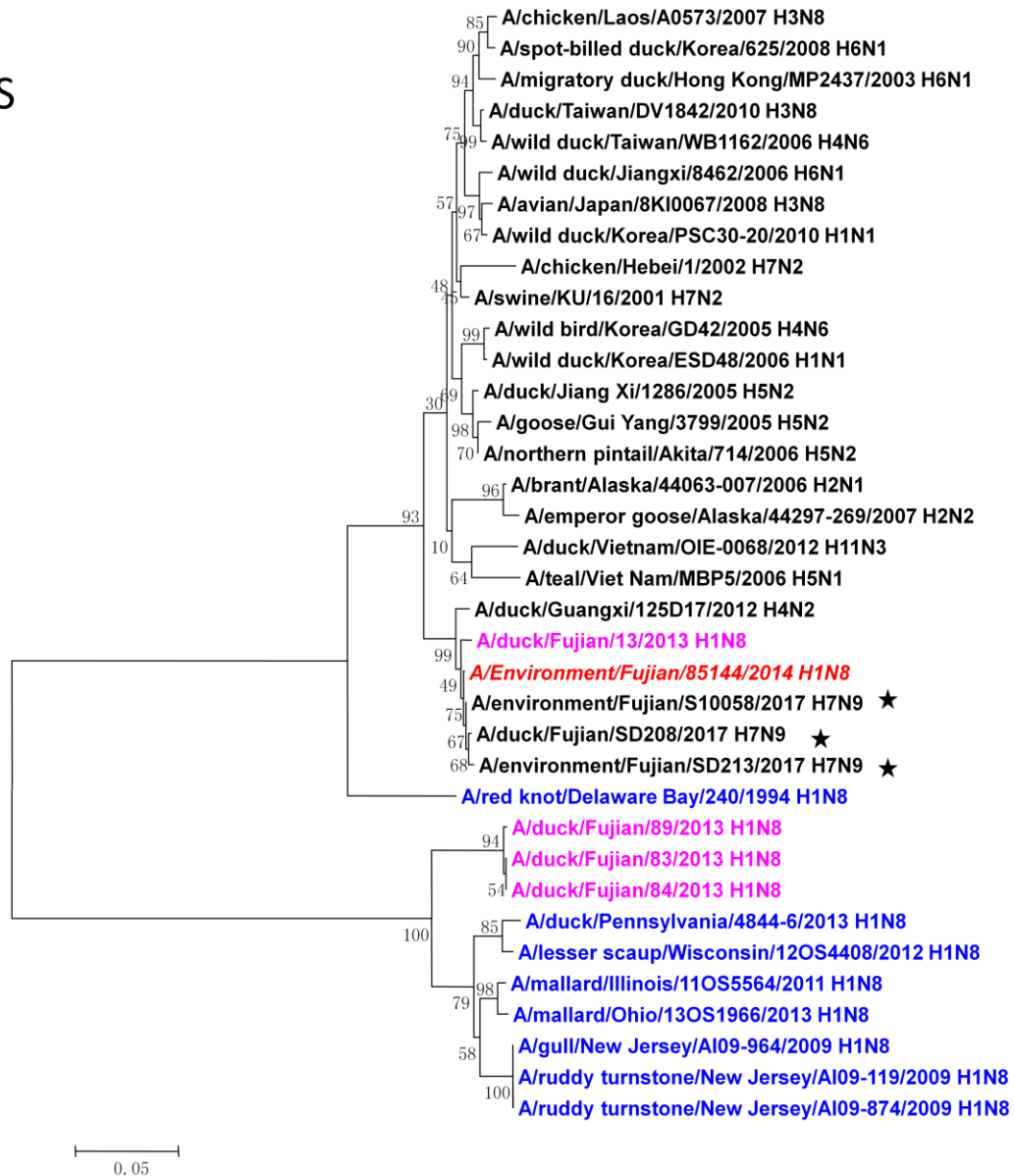

Supplement: Supplementary file 1 — Figure S1. Phylogenetic analysis of six internal genes of A/Environment/Fujian/85144/2014(H1N8). Phylogenetic analysis of six internal genes, including PB2, PB1, PA, NP, M, and NS. All sequences were downloaded from GenBank. The maximum likelihood method was used to construct the phylogenetic trees. The virus strains are marked with different colours. Red represents the A/Environment/Fujian/85144/2014(H1N8) strain in this study, blue represents other H1N8 subtype viruses, pink represents all available previous H1N8 subtype viruses sequence from China, black represents viruses of other subtypes, and the black star indicates the HPAI H7N9 viruses isolated in 2017 and 2018. (PDF 1538 kb) [file 12879_2019_4079_MOESM1_ESM.pdf]
